# Ethnobotanical Study of Wild and Semi‐Wild Edible Plants in Addi Arkay District, Northwestern Ethiopia

**DOI:** 10.1155/tswj/6632779

**Published:** 2026-03-20

**Authors:** Worku Misganaw, Getinet Masresha, Ermias Lulekal, Asmamaw Alemu, Daniel Tadesse

**Affiliations:** ^1^ Department of Biology, Debark University, Debark, Ethiopia, dku.edu.et; ^2^ Department of Biology, University of Gondar, Gondar, Ethiopia, uog.edu.et; ^3^ Department of Plant Biology and Biodiversity Management, Addis Ababa University, Addis Ababa, Ethiopia, aau.edu.et; ^4^ Department of Forestry, University of Gondar, Gondar, Ethiopia, uog.edu.et; ^5^ Department of Plant Sciences, University of Gondar, Gondar, Ethiopia, uog.edu.et

**Keywords:** food security, indigenous knowledge, nutraceutical, sociodemographic factors, use values

## Abstract

The exploration of wild and semi‐wild edible plants (WEPs) has received global attention as valuable alternative food sources. This study explored and documented the indigenous knowledge of WEPs within the Addi Arkay District of Ethiopia. Ethnobotanical data were collected from 385 informants, including 355 general informants selected through systematic random sampling and 30 purposively selected key informants. The analysis employed the frequency of citations, UVs, preference ranking, DMR, Jaccard similarity index (JSI), *t*‐tests, and one‐way ANOVA. In the study area, 42 species belonging to 28 families were identified as WEPs. The *t*‐test and one‐way ANOVA results revealed that indigenous knowledge of WEPs in the study area was significantly influenced by sociodemographic (age, gender, education, and informant types) and socioeconomic factors (occupation). Greater knowledge among elders underscores the need to preserve and transmit this wisdom to younger generations. Moraceae was the dominant family (9.5%), shrubs represented the largest growth form (42.9%), and fruits were the most commonly used plant part (64.3%). Most species (26, 61.9%) were collected from natural forests. Fruits were the most commonly used (61.9%). The most common preparation and consumption methods were fresh and raw (71.43%). The frequency of citations, use values, and JSI ranged from 83.38% to 4.16%, 0.97% to 0.03%, and 9.68%–95.83%, respectively. *Diospyros mespiliformis* was the most preferred WEP, with *Cordia africana* and *D. mespiliformi*s being the most common multipurpose species. The peak collection occurred from September to November, and *Ziziphus spina-christi* and *D. mespiliformis* were the most marketable species. Deforestation, fuelwood extraction, and house construction represent the primary threats to WEPs. The WEPs in the study area support food security, nutrition, livelihood, and cultural values. Future research should focus on nutritional and phytochemical analyses, conservation, domestication, and the sustainable commercialization of nutraceutical WEPs.

## 1. Introduction

Throughout history, approximately 40,000–100,000 plant species have been used for various purposes, representing approximately 5% of the world’s total plant species [1]. Of these, approximately 30,000 species are recognized as edible, with approximately 7000 having been cultivated or collected as food at different times [2]. This global diversity is reflected in Ethiopia, where the flora includes an estimated 6027 higher plant species, of which 647 (10.74%) are endemic [3]. Nearly 11.27% (679 species) of this diverse genetic resource have been identified as wild edible plants (WEPs) that supplement food sources across various regions and conditions in the country [4]. Food insecurity is a pressing global concern that drives the search for alternative and resilient food sources [5]. Among these, WEPs have gained recognition as important dietary supplements for growing populations [6]. These plants provide essential nutrients, including proteins, carbohydrates, lipids, minerals, vitamins, and bioactive compounds, such as antioxidants, phenols, flavonoids, and fibers [7]. Furthermore, evidence suggests that WEPs may be healthier than processed foods and safer alternatives to cultivated vegetables, which can contain high levels of agrochemical residues [5]. Consequently, the perception of WEPs as mere “famine foods” should be reconsidered, and these species should be integrated into daily diets as staples or supplementary foods.

In Ethiopia, recurrent droughts, civil conflicts, rapid population growth, low agricultural productivity, and rising living costs exacerbate food insecurity, thereby increasing the importance of WEPs [8]. According to the FAO‐WFP report, Ethiopia is classified as a hunger hotspot, with approximately 13 million individuals urgently needing humanitarian food assistance [9]. Given their nutritional and nutraceutical benefits, WEPs are promising alternatives for alleviating food crises. However, these resources face multiple threats, including agricultural land expansion, human settlement, forest burning, charcoal and timber production, deforestation, construction, the spread of exotic invasive plant species, and climate change, all of which contribute to local extinction [10].

Beyond emergency food provision, WEPs contribute significantly to dietary diversity, malnutrition reduction, and alternative food supply during geopolitical instability and famine [11]. These plants are valued over cultivated crops due to their genetic diversity, minimal selective breeding, low‐input requirements, drought tolerance, pest resistance, biochemical diversity, accessibility, affordability, and limited management requirements [12]. In food‐insecure rural households, WEPs account for up to 27.3%–35% of food intake during lean seasons [13], highlighting their importance as both emergency and supplementary foods.

In addition to their nutritional benefits, WEPs provide critical ecosystem services, including maintaining genetic diversity, supporting agroforestry in dryland regions, providing habitats for diverse organisms, rehabilitating degraded lands, pollinator support, soil stabilization, enhancing soil and water quality, and contributing to climate change mitigation and adaptation [14]. These ecological functions align with Ethiopia’s National Biodiversity Strategy and Action Plan (EBSAP), which prioritizes the conservation and sustainable use of neglected and underutilized species (NUS) [15]. Moreover, global frameworks such as the Food and Agriculture Organization’s (FAO) initiative on NUS emphasize WEPs as essential for achieving resilient food systems [16]. Promoting sustainable utilization and conservation of WEPs offers an effective strategy for advancing Sustainable Development Goals (SDGs), specifically those related to zero hunger (SDG 2), good health and well‐being (SDG 3), and climate change (SDG 13) [17, 18].

Despite their significance, WEPs are often overlooked compared with domesticated crops. In addition to their nutritional and ecological roles, WEPs contribute significantly to rural livelihoods. Beyond their nutritional and ecological roles, WEPs substantially contribute to rural livelihoods, serving as sources of income, traditional medicine, forage, fuelwood, construction materials, honey production, and natural detergents, reflecting their multifunctional role in sustaining rural households and local economies [19]. Furthermore, they preserve cultural heritage and indigenous knowledge, often concentrated among the elderly and integral to local identity [20]. However, institutional and policy limitations, including minimal integration into agricultural extension programs, absence from germplasm conservation efforts, and limited market incentives, restrict their effective utilization and accelerate the erosion of indigenous knowledge, particularly as younger generations adopt modern lifestyles [21].

Several ethnobotanical studies across Ethiopia have documented the importance of WEPs in supporting food security, nutrition, and traditional knowledge. For instance, 36 edible plants were recorded in Quara [22] and Tach Gayint [23] districts of the Amhara region, 62 in Kofale and Heban‐Arsi [24], 34 in Berek Natural Forest [8] of Oromia, 66 in Derashe and Kucha [13] of southern Ethiopia, 59 in Raya‐Azebo [25] of Tigray, 16 in Yalo District of Afar [26], 77 in Bullen [27], and 54 in Dibatie [28] of Benishangul‐Gumuz. Despite this extensive documentation, WEPs in Ethiopia remain largely unexplored, with approximately 8.4% of districts currently recorded [29]. Ethnobotanical knowledge is predominantly transmitted orally and concentrated among older generations, rendering it vulnerable to loss and contributing to the decline of information regarding species that support community nutrition and health [30]. Nevertheless, WEPs continue to play a crucial role in subsistence, climate change adaptation, and food security, offering resilient, low‐input alternatives to conventional crops [31]. Integrating WEPs into agricultural systems can enhance dietary diversity, improve nutrition, promote environmental sustainability, and strengthen livelihood diversification [11], particularly for low‐income households such as those in Addi Arkay District, while simultaneously safeguarding indigenous knowledge and biodiversity for future generations.

Addi Arkay District was selected for its diverse agroecology, cultural richness, and vulnerability to drought. The district is home to a rich diversity of WEPs, supported by the Waldeba Monastery Forest, which is the largest remnant of natural forest in the study area. However, like many areas in northern Ethiopia, Addi Arkay experiences rapid land‐use changes, deforestation, and increasing reliance on external food aid, underscoring the need for local‐level documentation and conservation efforts. These unique socioecological conditions present an opportunity to generate insights that can inform policies related to conservation, sustainable use, and food security at both local and national levels. To address these gaps, this study focuses on four key research questions: (i) How do sociodemographic and socioeconomic factors influence indigenous knowledge of WEPs? (ii) What is the diversity of WEPs and how are they utilized by local communities? (iii) What are the potential nutraceutical, marketable, and threatened WEPs for future nutritional analysis and conservation? (iv) How can WEPs be compared with previously published studies in Ethiopia to uncover new data on consumed species and cultural variation?

## 2. Methods and Materials

### 2.1. Description of the Study Area

**FIGURE 1:**
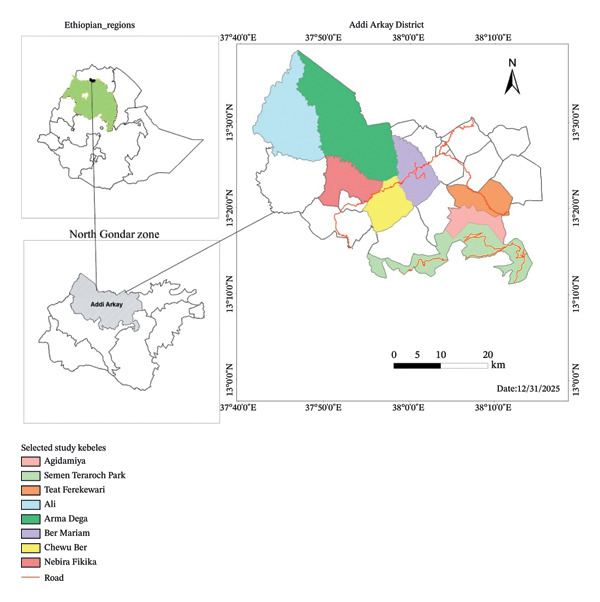
Map of the study area showing selected kebeles.

The study was conducted in the North Gondar Zone of Ethiopia’s Amhara Region, located approximately 837 km northwest of Addis Ababa. Geographically, the area lies between 13°10′0″–13°40′0‴N latitude and 37°40′0″–38°20′0‴E longitude (Figure 1).

The agroecological zones of the Addi Arkay District are predominantly lowlands (67.4%, 500–1500 m), followed by midlands (23.4%, 1500–2500 m), highlands (7.3%, 2500–3200 m), and upper highlands (1.9%, > 3200 m). The district exhibits a unimodal rainfall pattern, with an average annual precipitation of 1777 mm. The mean annual temperature is 22.8°C, with minimum and maximum averages of 11.9°C and 37.4°C, respectively [32]. In 2024, the total population of Addi Arkay District was 125,908, consisting of 63,865 males and 62,043 females. The religious affiliations were Orthodox Christians (89.5%) and Muslims (10.5%). Ethnically, the population was predominantly Amhara (97.6%) and Tigre (2.1%), with other groups accounting for 0.3%. Linguistically, Amharic is the primary language, spoken by 98.02% of the population, followed by Tigrinya (1.8%) and other languages (0.18%).

### 2.2. Reconnaissance Survey and Informant Selection

Following a reconnaissance survey conducted from July 5 to July 30, 2024, and consultations with district officials and community elders, eight kebeles (subdistricts) were selected using a stratified random sampling approach. The selection was guided by the district’s major agroecological zones and sociocultural settings to ensure representative coverage of indigenous and local knowledge of wild and semi‐WEPs. The chosen kebeles represent 36.36% of the 22 kebeles in the Addi Arkay District, providing adequate representation of the study area.

The informant sample size was determined using Cochran’s formula (*n* = *N*/[1 + *N*(*e* ^2^) [33], where *n* is the sample size, N is the total number of households in the district, and *e* is the margin of error (0.05)^2^ at the 95% confidence level. The number of informants from each kebele was determined using the following formula: number of households in one kebele/total number of households in all kebeles) × total sample size. Accordingly, 385 informants (355 general and 30 key) were selected, with a sex distribution of 229 men and 156 women (Supporting file 1). General informants were chosen through systematic random sampling, whereas key informants were purposively selected based on recommendations from local elders and leaders for their extensive knowledge of WEPs. Traditional school (church) teachers and students were categorized into the literate group (ability to read and write) for the education status variable. Fortunately, informants of traditional school (church) teachers and students were found to attend formal education and were thus classified into the formal educational level classification.

The socioeconomic status of informants was classified according to the income‐based categories of the local community. This classification considered income derived from farming, sales of cash crops and livestock, land rent, and other livelihood activities, guided by local economic standards, estimated annual household income, and community consensus. Based on these criteria, households with an annual income of less than USD 1000 were categorized as “poor,” those earning between USD 1000 and 10,000 as “medium,” and those with incomes exceeding USD 10,000 as “rich.” This categorization is consistent with the national rural livelihood and poverty assessment guidelines [34, 35]. The study sites were predominantly rural, encompassing both agrarian villages and monastic settlements, to capture the diversity of traditional ecological knowledge among farmers, ascetics (monks and nuns), and other occupational groups. This design ensured the representation of both secular and monastic communities that maintained long‐standing traditions in the use and management of WEPs.

### 2.3. Data Collection and Specimen Identification

Ethnobotanical data were collected using established protocols, including semistructured interviews, focus group discussions (FGDs), and guided field walks [36, 37]. Data collection was primarily based on semistructured interviews, which provided a flexible framework for in‐depth exploration of specific topics while enabling informants to share their personal experiences and a nuanced understanding of WEPs in a conversational context. A diverse range of informants were engaged in gathering detailed data on various aspects of WEPs, including local plant names, parts utilized, modes of consumption, harvesting months, diversity of usage, nutraceutical plants, and threats to WEPs. Interviews were predominantly conducted in Amharic, the common language in the study area, to ensure clarity and facilitate open dialogue. Following the interviews, all documented data were meticulously translated into English for subsequent analysis.

FGDs were employed to complement individual interviews, providing a platform for collective knowledge sharing and community dialogue. This methodological approach facilitated the exchange of ideas and experiences related to wild and semi‐WEPs, thereby elucidating both community consensus and perspectives regarding usage diversity and nutraceutical properties. FGDs also served to verify and enrich the data collected during the interviews, adding depth to subsequent analyses. A total of 12 informants (six men and six women) participated in each FGD. To encourage open communication and address potential gender‐specific perspectives, such as communication styles, emotional expression, shyness, and fear of judgment, FGDs were conducted separately for men and women participants [38]. These sessions were conducted at a convenient location within the community and meticulously documented through detailed note‐taking for subsequent analysis.

Prior to data collection, informed consent was obtained from all participants, and the study was conducted in accordance with the ethical guidelines of the International Society of Ethnobiology. Specimen identification was based on the Flora of Ethiopia and Eritrea and was assisted by senior botanists. Pressed specimens were deposited in the University of Gondar Herbarium. Scientific name updates were verified using World Online Plants, whereas the conservation status of endemic plants was identified using the IUCN Red List. To ensure the validity and reliability of the ethnobotanical data, a triangulation technique was employed, integrating multiple data collection methods and diverse informant perspectives to cross‐validate findings, mitigate bias, and enhance the robustness of the conclusions [39].

### 2.4. Quantitative Ethnobotanical Data Analyses

Basic ethnobotanical data were systematically analyzed and are presented in tables and graphs. Comparisons of informant knowledge of wild and semi‐WEPs were assessed across various factors using SPSS Version 25. Two‐tailed independent sample *t*‐tests were applied to detect differences between two‐category variables, whereas one‐way analysis of variance was used to evaluate variations among three or more categories. For variables with multiple levels, post hoc tests were performed, including Tukey’s honestly significant difference (HSD) and least significant difference (LSD), and were conducted to identify specific group differences. Additionally, quantitative analyses included the frequency of citation, use values, preference ranking, direct matrix ranking (DMR), and Jaccard similarity index (JSI).

### 2.5. Frequency of Citation

The frequency of citations for the popularity of WEPs among local informants was calculated by the following [40] formula:

(1) FC %=Number of informant citations for each speciesTotal number  of  informants interviewed×100,

where FC (%) = frequency of citation in percent.

### 2.6. Use Value

The value following [41] was used to determine the relative cultural importance of each individual species:

(2) UVs=∑inUisns,

where UV_*s*_ = use value of species *S*, *U*_*i* *s*_ = number of uses of species *S* according to informant *i*, and *n*_*s*_ is the total number of key informants interviewed.

### 2.7. Preference Ranking

A preference ranking exercise, following [37], was conducted to evaluate 10 WEPs for sweetness and eight WEPs for marketability. Ten randomly selected informants ranked the preselected species based on personal preference and perceived community importance. The scores ranged from 10 (most preferred) to 1 (least preferred) for sweetness, and the total score for each species was summed to determine the overall ranking.

### 2.8. DMR

Following [37, 42], DMR was used to identify WEPs under the greatest pressure and their respective threats. Fifteen key informants evaluated 12 multipurpose WEPs across seven use categories (agricultural tools, buildings, medicine, fodder, food, fuel, and furniture). Informants assigned the following values for each use category (5 = best, 4 = very good, 3 = good, 2 = less used, 1 = least used, and 0 = not used). Subsequently, the values assigned to each usage category and species were averaged and ranked.

### 2.9. JSI

JSI% was employed to assess cross‐cultural similarities and differences in the use of wild and semi‐WEPs documented in this study compared with previous ethnobotanical studies conducted across Ethiopia. This comparison aimed to determine the degree of overlap in species utilization among communities inhabiting different agroecological and sociocultural settings. Reference studies were selected based on two main criteria: (i) those conducted in regions representing diverse agroecological and sociocultural contexts and (ii) those providing comprehensive documentation of WEPs with sufficient ethnobotanical details to allow meaningful cross‐comparison. JSI was calculated as follows [43]:

(3) JSI%=ca+b−c×100,

where *a* = number of WEPs only in this study, *b* = number of WEPs only in the previous study, and *c* = number of WEPs in common.

## 3. Results

### 3.1. WEPs’ Indigenous Knowledge of Informants

A comprehensive investigation into the determinants of indigenous knowledge of WEPs within the Addi Arkay District revealed significant differences across various parameters (Table 1). Key informants (*n* = 30) exhibited a substantially higher mean number of plants mentioned (18.07 ± 2.70) than general informants (*n* = 355) (8.83 ± 3.12, *p* ≤ 0.001). Similarly, men’s informants demonstrated higher knowledge levels (10.11 ± 4.38) than women’s (8.74 ± 3.08, *p* ≤ 0.001). Knowledge of WEPs was positively correlated with age, with individuals aged ≥ 60 years scoring the highest (11.27 ± 4.67) compared to the 40–59 (9.12 ± 3.00) and 20–39 (8.84 ± 3.82, *p* ≤ 0.001) age groups. In relation to education status, illiterate informants (9.84 ± 3.97) possessed notably greater knowledge than literate individuals (7.97 ± 3.54, *p* ≤ 0.001). Occupational diversity emerged as a key determinant, with farmers (9.77 ± 3.93), individuals engaged in multiple occupations (9.67 ± 3.88), and ascetics (9.65 ± 4.19) demonstrating significantly elevated mean number of plants mentioned compared to those employed in formal jobs (7.18 ± 3.71) and merchants (6.91 ± 1.81, *p* ≤ 0.017). Similarly, income level correlated inversely with knowledge, with individuals classified as poor exhibiting greater knowledge (9.93 ± 4.31) than those classified as rich (8.12 ± 3.48, *p* ≤ 0.013). However, educational level, marital status, ethnic background, religion, distance from the town, and transport access had no significant effect on the mean number of plants mentioned by the informants.

**TABLE 1:** WEPs’ indigenous knowledge variation among informants (*n* = 385).

Parameter	Informant categories	Number of informants (*n*)	Mean ± SD (number of WEPs mentioned)	*F*‐statistic	*p* value
Sociodemographic parameters					
Nutritional experience	Key informants	30	18.07 ± 2.70	246.556	0.000^∗^
	General informants	355	8.83 ± 3.12		
Gender	Men	229	10.11 ± 4.38	11.442	0.001^∗^
	Women	156	8.74 ± 3.08		
Age (years)	20–39	166	8.84 ± 3.82^c^	13.563	0.000^∗^
	40–59	120	9.12 ± 3.00^b^		
	≥ 60	99	11.27 ± 4.67^a^		
Education status	Illiterate (unable to read and write)	326	9.84 ± 3.97	11.50	0.001^∗^
	Literate (able to read and write)	59	7.97 ± 3.54		
Education level	1–6	26	8.35 ± 3.451^a^	0.367	0.694
	7–12	24	7.67 ± 3.47^b^		
	> 12	9	7.33 ± 4.15^c^		
Marital status	Single	92	8.99 ± 3.96^d^	0.851	0.467
	Married	283	9.74 ± 4.00^c^		
	Widowed	7	9.29 ± 2.56^b^		
	Divorced	3	10.00 ± 1.00^a^		
Ethnic background	Amhara	365	9.62 ± 4.00^a^	1.187	0.306
	Tigray	15	8.40 ± 2.77^b^		
	Agew	5	7.80 ± 3.35^c^		
Religion	Orthodox	357	9.64 ± 4.04	2.445	0.119
	Muslim	28	8.43 ± 2.59		

Socioeconomic parameters					
Occupation	Farmer	295	9.77 ± 3.93^a^	3.048	0.017^∗^
	Merchant	11	6.91 ± 1.81^e^		
	Employed (in a formal job)	17	7.18 ± 3.71^d^		
	Ascetics (Monk/nun)	56	9.65 ± 4.19^c^		
	Two or more occupations	6	9.67 ± 3.88^b^		
Transport access	Accessible (road and car)	105	9.57 ± 4.32	0.014	0.906
	Not accessible (nor road and car)	280	9.51 ± 2.82		
Income level	Poor (estimated annual income less than $1000)	270	9.93 ± 4.31^a^	4.420	0.013^∗^
	Medium (estimated annual income of $1000–$10000)	89	8.84 ± 2.57^ab^		
	Rich (estimated annual income greater than $10000)	26	8.12 ± 3.48^b^		
Distance from town (km)	Less than 3 km	37	9.68 ± 3.12^b^	1.299	0.274
	3–5 km	25	10.68 ± 3.34^a^		
	6–8 km	45	8.76 ± 3.28^d^		
	More than 8 km	278	9.56 ± 4.19^c^		

*Note: n* = number of informants, *p* value = probability value, *F*‐statistic = Fisher’s *F*‐statistic.

Abbreviation: SD = standard deviation.

^∗^Significant difference for the parameters at *p* < 0.05.

### 3.2. WEPs’ Diversity

In this study, 42 WEPs were distributed across 33 genera and 28 families (Supporting file 2). The family Moraceae was represented by four species, accounting for 9.5% of the total WEPs encountered. This was followed by Dioscoreaceae and Fabaceae, each comprising three species (7.1%). The families Acanthaceae, Apocynaceae, Malvaceae, Polygonaceae, Rhamnaceae, Rosaceae, and Solanaceae were each represented by two species (4.8%). The remaining families contained one species, representing 2.4% of the total population. This study identified two endemic WEPs, *Urtica simensis* Hochst. ex A.Rich., and *Acanthus sennii Chiov.*, which have a least concern conservation status. Additionally, 19 species (45% of the total WEPs) were identified as nutraceutical plants (Table 2).

**TABLE 2:** Nutraceutical plants in Addi Arkay District of Ethiopia [32].

Nutraceutical WEPs	Use	Ailment treated	PU	CPU	MP	RA
*Capparis tomentosa*	Hu	Toothache	R	F	Chew the root along with *Zingiber officinale* and hold it on the teeth for a few minutes	O
		Cough	R	F	Grind it with the root of *Rosa abyssinica*, then burn the mixture and inhale the smoke	N
		Evil sprite	R	D	Burn the root and use the smoke to fumigate the house	Ot

*Carissa spinarum*	Hu	Evil sprite	R	D	Burn the root and use the resulting smoke for fumigation	N
	Li	Eye disease	L	F	Squeeze the leaf to extract the juice, then apply a few drops to the eyes	Op

*Corchorus olitorius*	Hu	Back pain	L	F	Collect the young leaves and eat the fresh leaf or cook and eat	O

*Cordia africana*	Hu	Gastritis	F	F	Consume the fruit directly	O
		Scorpion bite	R	F	Chewing the root and swallowing	O
		Hemorrhoid	SB	D	Grind the stem bark of it with the root bark of *Rydingia integrifolia*, mix with honey or butter, and smear	T
		Tape worm	F	F	Swallow the fruit	O

*Datura stramonium*	Hu	Toothache	S	D	Put the dry seed onto the fire to create smoke for fumigation, or hold the seed with your teeth	O
		*Tinea capitis* (quaqucha)	L	F	Squeeze the leaf and cream it on head	T

*Diospyros mespiliformis*	Hu	Ringworm	F	F	Paint the affected area with the fruit	T

*Dovyalis abyssinica*	Hu	Boil (bugungi)	L	F	Crush the leaf and paste it on the boil	T

*Ficus sycomorus*	Hu	Scorpion bite	SB	F	Chew and swallow the liquid	O
		Impotency	La	F	Smear the latex on the surface of the penis	T
		Hepatitis	L	F	Squeeze the leaf, then drink it with honey or buttermilk	O

*Gardenia ternifolia*	Hu	Impotency	SB	F	Grind the ingredients and mix them with Hydromel (honeyed wine), then drink the mixture	O

*Grewia ferruginea*	Hu	Retained placenta	SB	F	Soak the stem bark in water, then drink the jelly‐like fluid	O
		Dandruff	SB	F	Wash the head with its soft bark	T
	Li	Retained placenta	SB	F	Soak the stem bark in water, then drink the jelly‐like fluid	O

*Opuntia ficus-indica*	Hu	Hemorrhoid	Fl	F	Mix the flower with honey and apply it gently around the anus	T

*Rosa abyssinica*	Hu	Stomachache	R	F	Socked in water then allow to drink watery extract	O

*Rumex abyssinicus*	Hu	Eye diseases	R	D	Dry and grind, then drink with tea	O
		Rheumatism (qurtimat)	R	F	Boil with Niger oil and sesame oil, then drink before meals	O
	Li	*Calf ascaris*	R	F	Crush the root and let it drench in water	O

*Rumex nervosus*	Hu	Tinea capitis (quaqucha)	L	D	Grind the leaves into a powder and dust it onto head	T
		Wound	L	D	Grind the leaves into a powder and dust it onto the wound and bind with green algae	T

*Tamarindus indica*	Hu	Cough	F	F	Eat the fruit	O
		Internal parasite	F	F	Soak the fruit in water and then drink the juice	O
		Diarrhea	F	F	Eat the fruit	O
		Hypertension	F	F	Soak the fruit in water, then mix it with chopped teff bread and eat	O

*Urtica simensis*	Hu	Gastritis	L	F	Boil the leaf and eat it regularly like cabbage or grind the leaf, squeeze, and drink before meals.	O

*Vachellia abyssinica*	Hu	Toothache	L	F	Chew the leaf and hold it on the teeth	O

*Ximenia americana*	Hu	Wound	SB	D	Grind the inner layer of the stem bark and dust it onto the wound	T
		Tonsillitis	L	F	Chew seven leaves and then apply the paste to the head	T

*Ziziphus spina-christi*	Hu	Dandruff	L	F	Squeeze the leaves to extract the fluid, then smear it	T

*Note:* Hu = human, Li = livestock, PU = part used (F = fruit, La = latex, Fl = flower, L = leaf, R = root, S = seed, SB = stem bark); CPU = condition of part used (F = fresh, D = dry); MP = method of preparation, RA = route of administration (N = nasal, O = oral, Op = optical, T = topical, Ot = other).

### 3.3. Growth Forms and Habitats of WEPs

**FIGURE 2:**
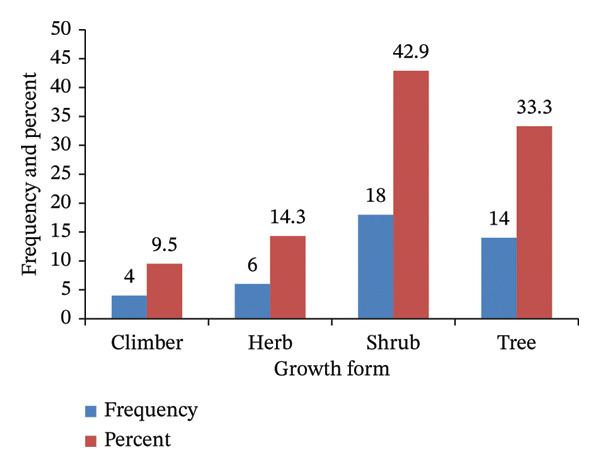
Growth form of wild edible plants in Addi Arkay District.

**FIGURE 3:**
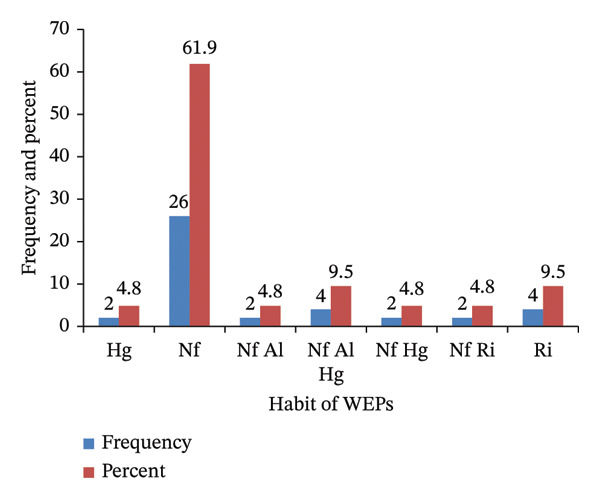
Habitat of wild edible plants in Addi Arkay District. Note: Hg = homegarden, Nf = natural forest, Al = arable land, Ri = riverine.

Documented WEPs comprise diverse life forms, including trees, shrubs, herbs, and climbers. Shrubs were the most represented group, with 18 species (42.9%), followed by trees with 14 species (33.3%) (Figure 2). WEPs were collected from various habitats, including natural forests, homegardens, riverine areas, and arable land. The majority of WEPs (26 species, 61.9%) were found in natural forests, while fewer species were found in riverine areas (four species, 9.5%) and homegardens (two species, 4.8%). The remaining WEPs were distributed across multiple habitats: four species (9.5%) in natural forests, arable land, and homegardens; two species (4.8%) in natural forests and arable land; two species (4.8%) in natural forests and homegardens; and two species (4.8%) in natural forests and riverine areas (Figure 3).

### 3.4. Edible Parts, Mode of Consumption, and Seasonal Availability of WEPs

In this study, eight distinct edible parts of WEPs were identified. These included fruit, flower nectar, gum, stem, root, tuber, corm, and leaf. The fruit was the most commonly utilized plant part, comprising 27 species (64.29%). Nectar from the flowers was utilized by four species (9.52%), and tubers by three species (7.14%). Edible stems and gums were represented by two species (4.76%) (Figure 4(a)).

FIGURE 4(a & b): Parts used and mode of consumption of wild edible plants.

**Figure:**
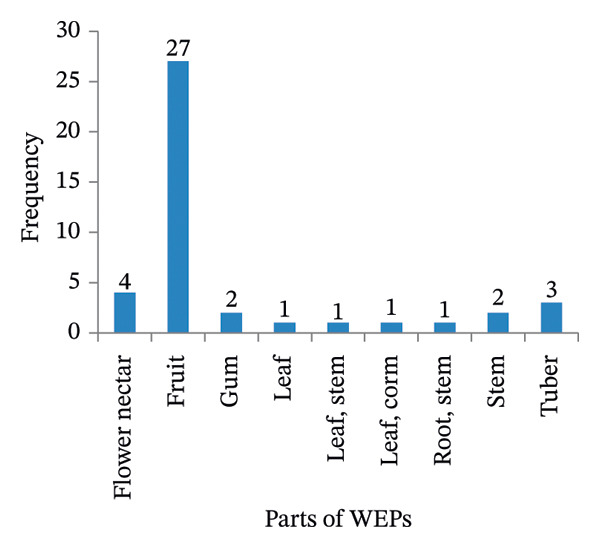
(a)

**Figure:**
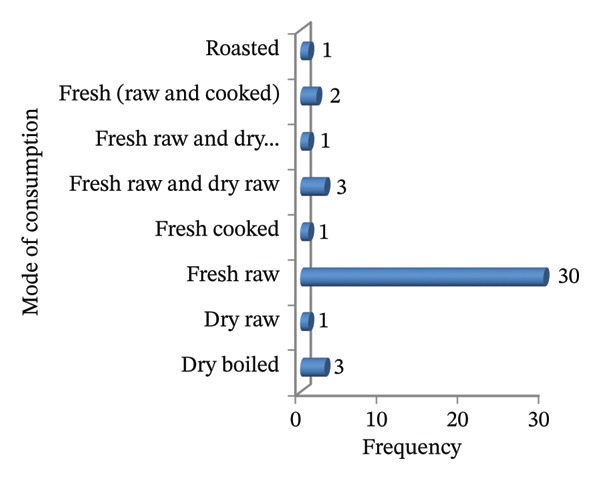
(b)

The predominant consumption methods were fresh and raw (30 species; 71.43%). A smaller proportion of the species were consumed fresh and dry (three species, 7.14%), while five species (11.9%) required processing, such as fresh cooking, boiling, or roasting. The remaining three species (7.14%) were consumed in various preparations, utilizing fresh and raw, dry boiled, or fresh and cooked methods, depending on the specific plant parts used (Figure 4(b)).

**FIGURE 5:**
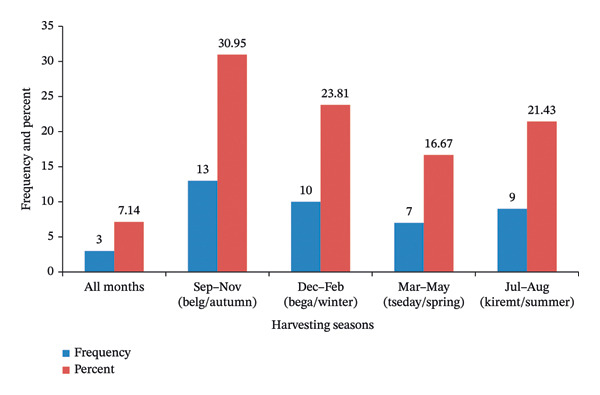
Harvesting months of wild edible plants.

Most WEPs were collected during the “Belg” (autumn) season, specifically from September to November, and 13 species (30.95%) were collected during this period (Figure 5). Ten species (23.81%) and nine species (21.43%) were collected during winter (December to February) and summer (July to August), respectively. Among the identified species, 39 were classified as seasonal, indicating that they were harvested during specific months. In contrast, three species were observed throughout the year: *Colocasia esculenta* (L.) Schott, *Vachellia seyal* var. *fistula* (Schweinf.) Kyal. & Boatwr., and *V. abyssinica* (Hochst. ex Benth.) Kyal. & Boatwr. Three primary techniques were predominantly used for gathering wild food plants: digging for tubers; plucking fruits, leaves, and gums; and collecting fallen seeds and fruits from the ground.

### 3.5. Ethnobotanical Ranking Indices of WEPs

#### 3.5.1. Occasions for Eating WEPs

Documented WEPs were categorized according to their consumption occasion (Supporting file 2). The majority of these species (34, 80.95%) served as supplementary food and were primarily consumed as snacks between meals, particularly by children and cattle herders. A smaller subset of WEPs (five species, 11.9%) was utilized as the main dish, while a limited number of species (three, 7.14%) were relied upon during periods of famine or drought, highlighting their critical role in food security during challenging times.

#### 3.5.2. Frequency of Citations

This study reveals significant variations in the frequency of WEP citations in terms of local community preferences and utilization. The percentage of citations ranged from 83.38% to 4.16% (Supporting 3). More than 50% of the informants cited 10 specific WEPs (23.81%) of the total species documented. The popularly cited species are *Diospyros mespiliformis* Hochst. ex A.DC., *Ziziphus spina-christi* (L.) Willd., *Cordia africana* Lam., *Carissa spinarum* L., *Ximenia americana* L., *Syzygium guineense* (Willd.) DC., *Ficus vasta* Forssk., *F. sycomorus* L., *Mimusops kummel* Bruce ex A.DC., and *Grewia ferruginea* Hochst. ex A.Rich (Table 3).

**TABLE 3:** Popular WEPs in Addi Arkay District of Ethiopia.

Scientific name	No. of informant citations	Percentage
Diospyros mespiliformis	321	83.38
Ziziphus spina‐christi	308	80.00
Cordia africana	297	77.14
Carissa spinarum	284	73.77
Ximenia americana	267	69.35
*Syzygium guineense*	249	64.68
*Ficus vasta*	235	61.04
*Ficus sycomorus*	234	60.78
*Mimusops kummel*	221	57.4
*Grewia ferruginea*	204	52.99

#### 3.5.3. Use Values of WEPs

The use values of WEPs in the Addi Arkay District ranged from 0.03 to 0.97 (supporting 4). *D*. *mespiliformis*, *C*. *africana*, *S. guineense*, and *F*. *vasta* had the highest usage values, reflecting their significant cultural importance within the local community. Conversely, *Ampelocissus schimperiana* (Hochst. ex A.Rich.) Planch., *Capparis tomentosa* Lam., *C*. *esculenta*, *Saba comorensis* (Bojer ex A.DC.) Pichon, and *Solanum villosum* Mill. were identified as WEPs with the lowest use values, indicating their low cultural relevance to the community.

#### 3.5.4. Preference Ranking

The preference ranking exercise for WEPs based on taste revealed that *D*. *mespiliformis* was the preferred species, followed by *C*. *spinarum*, *X*. *americana*, *C*. *africana*, and *Z*. *spina-christi* (Table 4).

**TABLE 4:** Preference ranking of wild and semi‐wild edible plants based on its sweet taste.

Plant species	Key informants (A‐J)										Total	Rank
	A	B	C	D	E	F	G	H	I	J		
*Carissa spinarum*	8	10	8	8	7	8	3	7	8	8	75	**2**
*Cordia africana*	1	7	7	4	9	6	9	2	1	10	56	**4**
*Diospyros mespiliformis*	10	8	2	5	10	9	8	10	10	6	78	**1**
*Ficus sycomorus*	3	1	3	3	4	5	6	3	2	1	31	**10**
*Ficus vasta*	4	6	10	2	6	10	4	6	3	3	54	**6**
*Grewia ferruginea*	2	3	5	1	3	2	1	5	5	5	32	**9**
*Mimusops kummel*	9	9	1	7	2	3	2	8	4	2	47	**8**
*Syzygium guineense*	5	5	6	9	8	1	7	1	6	4	52	**7**
*Ximenia americana*	7	4	9	6	5	7	10	4	9	9	70	**3**
*Ziziphus spina-christi*	6	2	4	10	1	4	5	9	7	7	55	**5**

*Note:* 10 = most preferred, 1 = least preferred. Bold values in the table are used to emphasize key information for readers.

#### 3.5.5. DMR for Multipurpose WEPs

The DMR of the multipurpose WEPs showed that *C*. *africana* was ranked first, followed by *D*. *mespiliformis*, *S*. *guineense*, *X*. *americana*, and *F*. *vasta* (Table 5). These multipurpose WEPs are currently being exploited and threatened primarily for fuel, building materials, food (particularly in relation to fruit harvesting), furniture, and fodder.

**TABLE 5:** Direct matrix ranking for multipurpose wild and semi‐wild edible plants.

Plant species	Use categories							Total	Rank
	Agriculture tools	Building	Medicinal	Fodder	Food	Furniture	Fuel		
*Carissa spinarum*	1	0	2	2	4	0	5	14	**9**
*Cordia africana*	4	5	4	5	4	5	5	32	**1**
*Diospyros mespiliformis*	4	5	3	4	5	5	5	31	**2**
*Ficus sur*	2	3	0	1	3	3	4	16	**7**
*Ficus sycomorus*	0	4	1	2	2	4	2	15	**8**
*Ficus thonningii*	1	1	0	5	2	1	2	12	**11**
*Ficus vasta*	1	4	0	2	3	3	5	18	**5**
*Grewia ferruginea*	1	1	2	5	2	0	2	13	**10**
*Phoenix reclinata*	0	3	0	2	2	1	1	9	**12**
*Syzygium guineense*	2	4	0	0	5	4	5	20	**3**
*Vachellia abyssinica*	3	5	1	2	1	4	1	17	**6**
*Ximenia americana*	1	3	4	1	3	2	5	19	**4**
Total	20	38	17	31	36	32	42		
Rank	**6** ^**th**^	**2** ^**nd**^	**7** ^**th**^	**5** ^**th**^	**3** ^**rd**^	**4** ^**th**^	**1** ^**st**^		

*Note:* 5 = best; 4 = very good; 3 = good; 2 = less used; 1 = least used; and 0 = not used. Bold values in the table are used to emphasize key information for readers.

#### 3.5.6. JSI

JSI analysis ranged from 9.7% to 95.8%, revealing vegetation type and using cultural variations in WEPs commonality between the study area and other regions (Table 6). The highest similarity, 95.83%, was observed between the Metema and Quara districts, followed by Chilga at 70.83% and Dibatie at 63.64%. In contrast, the lowest similarity was found between Yalo (15.00%), Konso Ethnic (11.02%), and Kara and Kwego semi‐pastoralist communities (9.68%). A higher JSI value revealed greater similarity in the use of WEPs between the study areas, reflecting similar vegetation types and plant use cultural interactions, whereas a lower value suggested distinct local knowledge and adaptation to specific ecological conditions.

**TABLE 6:** Jaccard’s similarity index of the current study compared with previous studies in Ethiopia.

Study areas (districts)	Number of WEPs report	*a*	*b*	*c*	JI (%)	References
Tach Gayint	36	28	22	14	38.89	[23]
Bullen	77	27	62	15	20.27	[27]
Dibatie	54	21	33	21	63.64	[28]
Quara	36	30	24	12	28.57	[22]
Mieso	41	31	30	11	22.00	[44]
Burji	46	31	35	11	20.00	[45]
Chilga	33	25	16	17	70.83	[46]
Soro	64	23	45	19	38.78	[47]
Arsi Robe	36	31	25	11	24.44	[48]
Midakegn	50	23	31	19	54.29	[1]
Raya‐Azebo	59	30	47	12	18.46	[25]
Simada	45	29	32	13	27.08	[49]
Yalo	16	36	10	6	15.00	[26]
Konso Ethnic	127	28	113	14	11.02	[50]
Derashe and Kucha	66	29	53	13	18.84	[13]
Awi Agäw community	39	27	24	15	41.67	[51]
Kofale and Heban‐Arsi	62	27	47	15	25.42	[24]
Metema and Quara	51	19	28	23	95.83	[52]
Kara and Kwego semi‐pastoralists	38	36	32	6	9.68	[53]
Berek Natural Forest	34	30	22	12	30.00	[8]

*Note:* The Jaccard similarity index (JSI) was computed using the following formula: JSI (%) = (*c*/(*a* + *b* − *c*)) × 100, where *a* represents the number of WEP species unique to this study, *b* denotes the number unique to the compared study, and *c* indicates the number of species shared by both studies. Higher JSI values reflected greater similarity in species use between the study areas.

#### 3.5.7. Marketability of WEPs

WEPs serve as vital food sources and contribute significantly to the income of local communities (Figure 6). Market surveys and informant interviews revealed that eight WEPs (19.05%) were sold as food in the local markets and towns. The most marketable WEPs were *Z. spina-christi*, *D. mespiliformis*, and *S. guineense* (Table 7).

FIGURE 6Some of the marketable WEPs in the Addi Arkay District. (a) *Tamarindus indica*. (b) *Ziziphus spina-christi*. (c) *Mimusops kummel*. (d) *Ximenia americana*.

**Figure:**
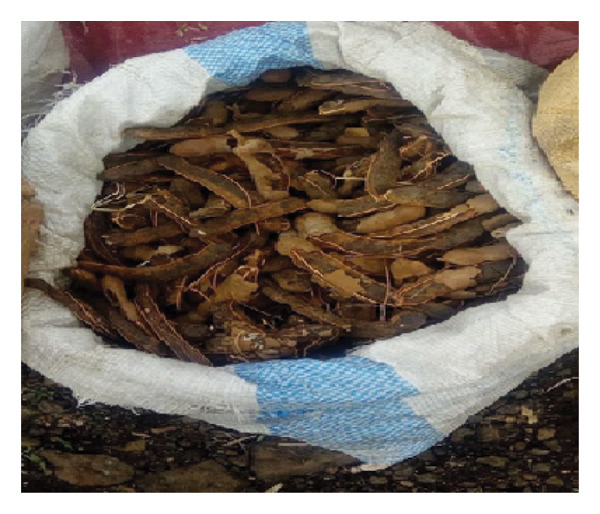
(a)

**Figure:**
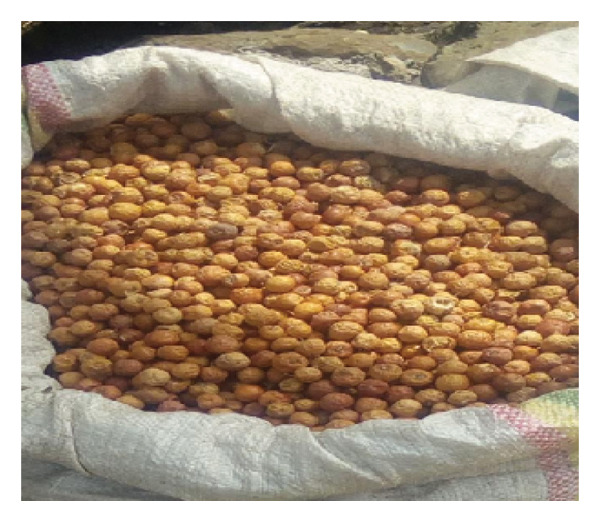
(b)

**Figure:**
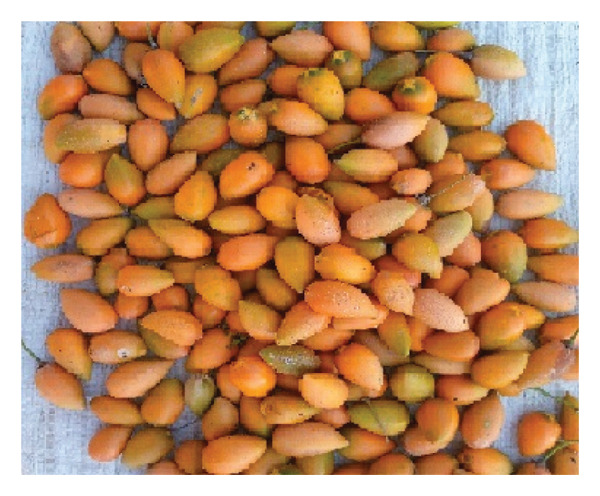
(c)

**Figure:**
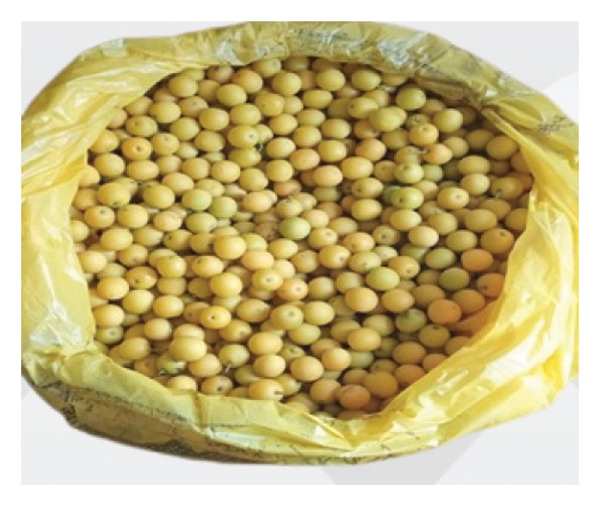
(d)

**TABLE 7:** Marketable WEPs in Addi Arkay District of Ethiopia.

Species name	Unit of measurement	Price in Ethiopian birr^∗^	Collector and seller		Rank
			Age	Sex	
*Cordia africana*	Small tin, larger tin	5/tin, 10/tin	Younger	Women	8^th^
*Diospyros mespiliformis*	Larger tin, kg	20/tin, 40/kg	All	Both sex	2^nd^
*Mimusops kummel*	Larger tin, number	20/tin, 1/pieces	All	Both sex	4^th^
*Strychnos innocua*	Number	1–1.5/pieces	Younger	Men	7^th^
*Syzygium guineense*	Plastic dish	15–20/dish	All	Both sex	3rd
*Tamarindus indica*	Number	5/3 pieces	Adult	Both sex	6^th^
*Ximenia americana*	Cup	5–7/cup	Adult	Men	5^th^
*Ziziphus spina-christi*	Small tin, larger tin	10, 20	All	Both sex	1^st^

^∗^1 US Dollar = 129.80 Ethiopian birr, local classification between 20 and 39 years old as younger and between 40 and 59 years of age as adult; ranks are given by the number of respondents.

#### 3.5.8. Threats and Conservation Practice for WEPs

The results showed that deforestation for agricultural land was the primary threat, followed by fuel (charcoal and fuelwood), and house construction (Table 8). Despite these significant threats, conservation efforts in Addi Arkay District remain limited. Current practices were terracing, growing WEPs in homegardens and arable land, fallow land, using WEP as live fences, and community participation in reforestation campaigns.

**TABLE 8:** Ranking exercise of threats to WEPs.

Threats of WEPs	Key informants										Total	Rank
	*k* − 1	*k* − 2	*k* − 3	*k* − 4	*k* − 5	*k* − 6	*k* − 7	*k* − 8	*k* − 9	*k* − 10		
Deforestation for agriculture	8	4	7	6	7	8	3	7	7	5	**62**	**1** ^**st**^
Use for food, forage, and medicine	2	5	6	3	1	7	2	8	3	3	**40**	**5** ^**th**^
Fuel (charcoal and fuel wood)	5	8	2	7	5	4	6	6	6	7	**56**	**2** ^**nd**^
Use for dead fence	4	2	1	5	8	3	7	4	2	1	**37**	**7** ^**rd**^
House construction	3	7	8	1	6	2	8	5	8	4	**52**	**3** ^**rd**^
Timber production	6	6	5	4	2	1	5	1	5	8	**43**	**4** ^**th**^
Agricultural tools	7	1	3	8	4	6	1	3	4	2	**39**	**6** ^**th**^
Drought	1	3	4	2	3	5	4	2	1	6	**31**	**8** ^**th**^

*Note:* 8 = value for most threatened and 1 = value for least threatened. Bold values in the table are used to emphasize key information for readers.

### 3.6. Novel Ethnobotanical Findings

This study documents the traditional preparation of Quarif, a sacred dish exclusively prepared for and consumed by monks and nuns at the Waldeba Monastery. “Quarif” is primarily made from three specific tuber plants, *Dioscorea praehensilis* Benth., *Dioscorea bulbifera* L., and *Dioscorea hispida* Dennst, along with unripe banana (Figure 7). Monks intentionally utilize unripe bananas rather than ripe ones, as they are less sweet and symbolically represent modesty, self‐restraint, and spiritual discipline. This preference for unripe bananas distinguishes monastic practices from secular habits, as ripe bananas are typically associated with sweetness and immediate consumption. Furthermore, the unripe form offers a longer shelf‐life after processing, a practical consideration for monastic communities that often rely on limited and infrequent food supplies. This cultural choice reflects monastic values that limit excess indulgence and emphasize simplicity, moderation, and detachment from material pleasures in daily life. These components play a crucial role in the cultural and spiritual significance of dishes in monastic communities.

FIGURE 7Ingredients for Quarif preparation at Waldeba Monastery. (a) *D*. *praehensilis* Benth.; (b) *D*. *bulbifera* L.; (c) *D*. *hispida* Dennst.; (d) *Musa spp*. (cultivated under‐ripe banana).

**Figure:**
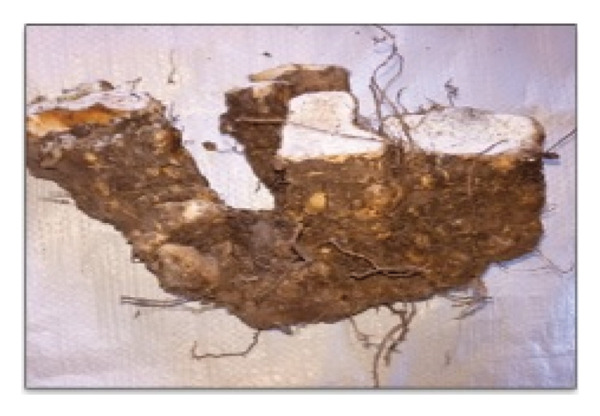
(a)

**Figure:**
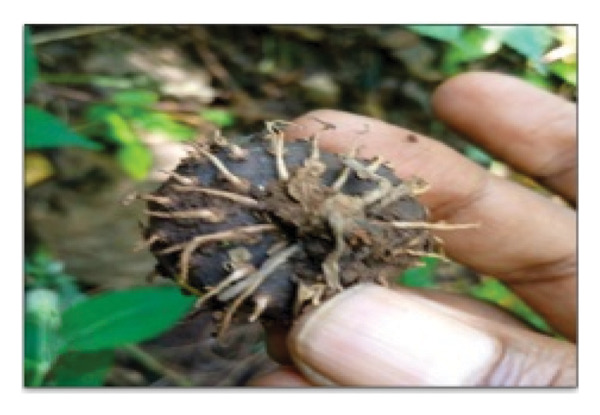
(b)

**Figure:**
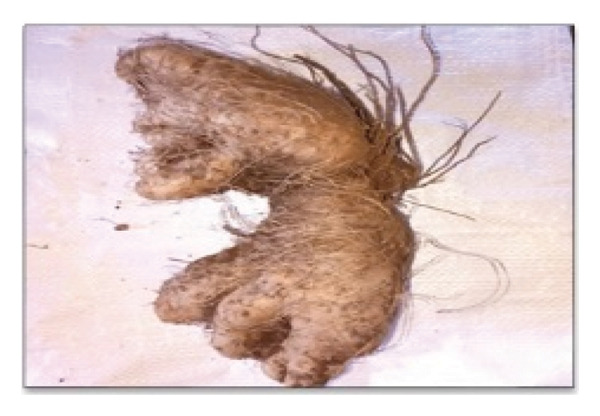
(c)

**Figure:**
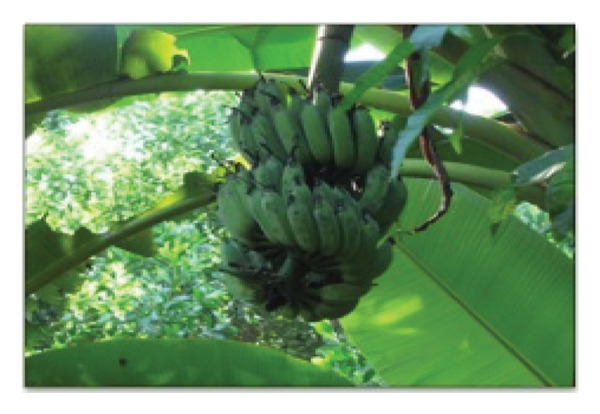
(d)

#### 3.6.1. Traditional Preparation of Quarif at Waldeba Monastery

*D*. *praehensilis* and *D*. *bulbifera* have similar preparation methods. The tubers were boiled, chopped, and sun‐dried. After drying, the samples were stored in clean containers to ensure longevity and maintain quality. This technique emphasizes simplicity and efficiency, allowing for easy storage and use in the preparation of “Quarif.” In contrast, the preparation of *D*. *hispida* (Tabile) is complex because of its naturally bitter taste. The process begins with boiling the tubers in water, followed by peeling away the hairy outer layer. The peeled tubers were then sun‐dried until sufficient dehydration was achieved. To remove bitterness, the dried tubers were soaked in water in a wicker container for 7 days. After soaking, the tubers were chopped and dried again under sunlight before being stored in clean and safe containers.

#### 3.6.2. Sustainability Concerns

*D. hispida* tubers are not attractive to wild animals, whereas the tubers of both *D.* praehensilis and *D. bulbifera* are frequently eaten by wildlife. This raises concerns about the availability of these species in their natural habitats. Thus, the monastic community has adapted traditional recipes by substituting these vulnerable tubers with cultivated under‐ripe bananas as a sustainable alternative that preserves Quarif’s cultural integrity while mitigating the overharvesting of wild *Dioscorea spp.* This practice exemplifies a proactive ethnoecological strategy within knowledge systems that dynamically addresses contemporary conservation challenges.

The preparation of “Quarif” using bananas involves a systematic approach beginning with the harvesting of under‐ripe bananas, followed by 3 days of sun‐drying. Subsequently, the bananas were boiled in water and dried for an additional day. The subsequent steps include peeling and chopping the bananas into pieces, which are then allowed to dry sufficiently under sunlight before being stored in clean and safe containers. This method ensures that the bananas can be preserved for up to 7 years without spoilage, making them a reliable alternative to the tubers traditionally used in “Quarif.”

#### 3.6.3. Serving Ascetics for “Quarif”

Before serving, the sacred meal known as “Quarif” is prepared by boiling the dried tubers and bananas in a 3:1 ratio of water to solids. This results in a porridge‐like consistency, which is consumed daily. “Quarif” is served once every 24 h, precisely at 9:00 a.m., using a standardized wooden spoon on a wooden dish. In addition to the banana porridge, ascetics also enjoy a drink made from Niger seed powder and linseed as a supplement to the “Quarif,” further enriching their daily nutritional intake. This careful preparation and ritual underscore the spiritual and cultural significance of Quarif in a monastic community.

## 4. Discussion

### 4.1. Indigenous Knowledge of the Informants on WEPs

The results indicated an unequal distribution of knowledge among different informant groups, with key informants exhibiting a significantly higher mean number of WEPs mentioned scores than general informants. This finding is consistent with those of studies conducted in the Midakegn District [1] and the Metema and Quara districts [52] in Ethiopia. Additionally, the higher knowledge levels observed among older generations can be attributed to their prolonged engagement with ecological practices and firsthand experiences in utilizing WEPs during food shortages. This trend is consistent with the findings of previous studies [49, 52] in Ethiopia and [39] in other regions.

The observed trend of men exhibiting higher scores in indigenous knowledge of WEPs than women was consistent with findings from other studies in Ethiopia [23, 54]. This may be attributed to sociocultural factors that restrict women’s access to WEP knowledge. Men generally have greater exposure to WEPs through activities such as cattle‐keeping, farming, and timber collection, facilitating more direct engagement with the local environment. However, this trend contrasts with a study in the Chelia District, where women demonstrated greater knowledge than men [55]. Additionally, the study revealed that illiterate individuals possessed a greater depth of WEP knowledge than their literate counterparts, with no significant increase in knowledge correlating with higher educational levels. This finding is consistent with research on WEPs in Ethiopia’s dryland ecosystems [21].

The findings revealed that occupational diversity significantly influenced knowledge of WEPs, with farmers demonstrating greater expertise than individuals from other occupations. This aligns with similar studies conducted in Ethiopia [44] and other regions [56]. The enhanced knowledge among farmers likely results from their direct and continuous interaction with the natural environment, which fosters a deeper understanding of available resources. Although household income levels are dynamic and may fluctuate over time, food insecurity can affect all groups. However, the effects are often more severe and prolonged among poorer households because of their limited livelihood options and restricted access to alternative food sources [1, 57]. Consequently, they rely heavily on wild and semi‐WEPs as essential sources of food and supplementary income. This reliance involves the frequent collection and sale of WEPs, which fosters closer interaction with the local environment and enables deeper practical knowledge of plant identification, seasonal availability, and diverse uses compared with medium‐ and high‐income households [6].

In contrast, wealthier households tend to depend more on cultivated or purchased foods, reducing their engagement with WEPs and leading to a gradual decline in traditional plant knowledge [22]. These findings align with other ethnobotanical studies in Ethiopia and elsewhere, which report that lower income households often maintain richer knowledge of WEPs as part of their adaptive strategies during food shortages [22, 58]. Similar patterns are evident in the Chilga District of northwestern Ethiopia, where economic constraints enhance reliance on local biodiversity for daily sustenance [46]. Overall, WEPs play an indispensable role in rural diets, particularly among the poor, serving as emergency food sources during crises and contributing significantly to food security and the resilience of subsistence livelihoods in developing regions [59].

### 4.2. WEPs’ Diversity

The 42 documented WEPs in the Addi Arkay District exceed those reported in the Arsi Robe (*n* = 36) [48], Chilga (*n* = 33) [46], Quara (*n* = 36) [22], Tach Gayint (*n* = 36) [23], Yalo (*n* = 16) [26], Aba’ala (*n* = 20) [60], and Sedie Muja (*n* = 33) [54] districts, suggesting greater reliance on WEPs in Addi Arkay. However, some studies have reported higher counts, such as Kofale and Heban‐Arsi districts (*n* = 62) [24], Bullen (*n* = 77) [27], Burji (*n* = 46) [45], Dibatie (*n* = 54) [28], Midakegn (*n* = 50) [1], and Chelia (*n* = 58) [55]. This variation in WEPs’ abundance may be attributable to cultural differences, vegetation types, and distinct agroecological zones within the study areas [61].

The dominance of the Moraceae family is attributed to a combination of ecological adaptability [44, 53] and cultural significance; the majority of *^∗^ Ficus* ^∗^ species are edible [62], and they exhibit a widespread geographic distribution across tropical and subtropical regions [63]. This result is in alignment with other WEP studies in the dryland ecosystem of Ethiopia [21], the Chelia District [55], the Metema District [64], and elsewhere [65]. In addition, Dioscoreaceae (yams) are intensively used because of their cultural and nutritional value in the Waldeba Monastery, which is regarded as a sacred food source. Yams are well‐known as crucial staple foods in tropical and subtropical regions, including the Sheko people of Ethiopia [66]. These energy‐rich tubers, derived from modified underground stems, are bulky and perishable, and their vegetative propagation underscores their vital role in local food security and agriculture [67].

### 4.3. Nutraceutical WEPs

The current study revealed a remarkable diversity of uses of WEPs that extend beyond their nutritional value as functional foods, aligning with recent findings across various regions of Ethiopia [25, 68] and elsewhere [69]. Diverse applications of WEPs, ranging from nutrition to medicine, have highlighted their multifaceted value in local communities. This diversity reflects a deep reservoir of traditional knowledge and reliance on wild plant resources, particularly in areas with limited access to domesticated foods and modern healthcare. WEPs are a source of essential nutrients but also possess bioactive compounds that contribute to health improvements, such as cholesterol‐lowering, antidiabetic, antiaging, antihepatotoxic, anticancer, antioxidant, antihypertensive, and anti‐inflammatory properties [70]. This integration enhances food and nutrition security, offering the dual benefit of supporting local livelihoods while simultaneously promoting community resilience in the face of changing environmental and economic conditions [71].

### 4.4. Growth Form and Habit of WEPs

Shrubs and trees dominate the growth forms of WEPs, enhancing their role in semiarid environments due to the year‐round availability of woody species, unlike herbaceous plants that thrive only during the rainy season. This finding aligns with previous studies on Ethiopia’s dryland ecosystems [21, 44, 72]. Woody species have developed adaptations to cope with high temperatures and water scarcity [73], making them crucial for food security and ecological value in the region. The collection of WEPs from diverse habitats—including natural forests, riverine areas, homegardens, and arable land—highlighted local biodiversity and adaptive strategies. A significant portion of the collection originated from the wild, corroborating previous reports [1, 44, 74]. Furthermore, the integration of WEPs from riverine ecosystems underscores their ecological importance by providing unique species that are vital for local diets and cultural practices [51]. Sustainable harvesting from these habitats reflects traditional ecological knowledge, which was crucial for maintaining biodiversity and promoting conservation efforts [49, 51]. Consequently, the collection of WEPs from multiple habitats supports local livelihoods and plays a critical role in conserving plant diversity in Ethiopia.

### 4.5. Edible Parts, Mode of Consumption, and Seasonal Availability of WEPs

This study revealed the diverse uses of the edible parts (seeds, stems, tubers, roots, leaves, gums, nectar of flowers, and fruits) of WEPs, enhancing food security by providing year‐round options. Fruits were the most commonly used part, consistent with various ethnobotanical studies in Ethiopia [27, 46, 52] and elsewhere [75]. The predominance of fruits in WEP consumption reflects their ecological accessibility and high nutritional value. Fruits were preferentially harvested for their ready‐to‐eat nature, requiring minimal processing and availability during dry periods. They are rich in soluble sugars and lipids, providing high caloric and micronutrient values [76]. This preference for fresh, raw food aligns with findings from various studies in Ethiopia [1, 45, 47, 52]. Consuming fresh WEPs maximizes nutrient intake, avoids heat‐induced vitamin loss, retains beneficial enzymes, and reflects traditional practices, offering a convenient means of accessing essential nutrients, particularly in resource‐limited settings [77].

The findings demonstrated that WEPs in the study area exhibited strong seasonal dependence, with the highest diversity being harvested during the “Belg” season (September–November). This is consistent with studies in Ethiopia [78, 79], which indicated that WEP availability peaked during the wet season owing to increased plant growth and fruiting. However, contrasting reports from the semiarid regions of southern Ethiopia [80] indicated that some communities relied more on drought‐resistant species during dry periods, suggesting that WEP harvesting patterns are highly context‐dependent and are influenced by agroecology and climate variability. Globally, similar seasonal trends have been observed in India [81] and sub‐Saharan Africa [82], where WEPs serve as critical famine foods during the lean season. Predominant harvesting techniques, including digging tubers, plucking fruits, and collecting fallen seeds, reflect indigenous knowledge systems that minimize ecological damage, as observed in Ethiopian studies [23] and global research [81]. Nevertheless, concerns remain regarding harvesting, particularly for slow‐growing species such as ∗ V. abyssinica ∗, which are threatened by deforestation [79].

### 4.6. Ethnobotanical Indices of WEPs

The frequent citation of species such as *D*. *mespiliformis* and *Z*. *spina-christi* suggests their cultural and nutritional importance, consistent with findings from other African regions where these species were valued for their drought resilience and nutrient density [83]. The reliance on a few key species during famine further emphasizes the critical safety net function of WEPs, a phenomenon documented globally in food‐insecure regions [84]. However, the limited use of WEPs as main dishes indicates a decline in traditional dietary practices, a trend observed in other studies [85]. This underscores the need for policies promoting WEP conservation and sustainable use to enhance food security.

The findings from the Addi Arkay District highlight the varying cultural significance of WEPs, with the highest use values underscoring their diverse cultural uses. This aligns with ethnobotanical research, which has shown that species with high use values exhibit deep cultural embeddedness, serving as key resources for food security, traditional medicine, animal feed, firewood, house construction, furniture, farm tools, and charcoal [8, 52]. Conversely, the low use values of certain species suggest limited cultural relevance, possibly because of taste preferences, limited availability, or a lack of traditional knowledge transmission [86].

The preference ranking results highlighted the culturally and sensory‐driven selection of WEPs in Ethiopia, with *D*. *mespiliformis* emerging as the most favored species because of its taste, followed by *C*. *spinarum* and *X*. *americana*. This finding aligns with global studies, demonstrating that taste significantly influences the selection and consumption of wild fruit [81]. Similar findings in Ethiopia [87] and other African regions [83] indicate that palatability drives the frequent use of WEPs, particularly among children and foraged snacks. The preference for these species suggests their potential for domestication or value‐added processing to enhance food security, as observed in studies promoting underutilized fruits for nutrition [11]. However, overharvesting of preferred species poses conservation challenges, necessitating sustainable management strategies [84].

The DMR underscored the multipurpose utility of WEPs, with *C*. *africana* and *D*. *mespiliformis* ranking the highest owing to their diverse uses in fuel, construction, food, and fodder. This reflects broader trends in Ethiopia [25, 28, 44, 52], where multipurpose WEPs are integral to rural livelihoods but are subject to overexploitation. The high demand for these species, particularly for nonfood uses such as fuel wood and timber, increased their vulnerability, as observed in studies on deforestation and WEP depletion [52]. The threat from destructive harvesting techniques, such as branch cutting for fruit collection, aligns with findings in [8, 23] and other regions [88], emphasizing the need for community‐based conservation and agroforestry integration to ensure sustainable use.

JSI analysis highlighted significant variations in WEPs composition between the study area and other Ethiopian regions, reflecting ecological and sociocultural influences. The high similarity between the Metema and Quara districts [52] suggests shared agroecological conditions, cultural practices, and geographical proximity. In contrast, low similarity with Yalo [26], Kara/Kwego communities underscored the impact of cultural isolation and specialized adaptations, such as Konso’s unique agroforestry systems and the Kara/Kwego’s pastoralist reliance on distinct species [53]. Globally, such disparities align with research, demonstrating that indigenous knowledge systems and environmental heterogeneity contribute to divergent patterns in WEP use [81]. These findings emphasize the need for localized conservation strategies and ethnobotanical documentation to capture region‐specific plant knowledge.

### 4.7. Marketability of WEPs

The findings revealed that WEPs contributed substantially to food security and local economies, with 19.05% of documented species traded in the markets. This aligns with global research demonstrating the dual role of wild foods in nutrition and income generation, particularly in rural communities [84]. The prominence of *Z*. *spina-christi*, *D*. *mespiliformis*, and *S*. *guineense* as top marketable species mirrored observations from other studies in Ethiopia [21, 46, 52] and similar contexts in sub‐Saharan Africa [83], where these species are valued for their taste and cultural significance.

The commercialization of WEPs presents both opportunities and challenges. On the one hand, it provides critical cash income, especially for women and marginalized groups, as documented in studies on nontimber forest products [89]. However, market demand has driven overharvesting, threatening species sustainability—a concern raised in studies on the wild fruit trade in Ethiopia [78] and elsewhere [90]. *Z*. *spina-christi*, often harvested destructively for its fruits, exemplifies this tension. To balance the economic benefits of conservation, policies should promote sustainable harvesting techniques and value‐added processing, as suggested by research on wild food commodification [11].

### 4.8. Threats and Conservation Practice for WEPs

Agricultural encroachment, identified as a primary anthropogenic driver of WEP depletion, was aligned with previous studies in Ethiopia [23, 46, 52]. The reliance on fuelwood and charcoal production constitutes a significant threat, corroborating research from Ethiopia [22, 78] and other developing regions, where energy demands exacerbate habitat degradation [91]. *C*. *africana* and *S*. *guineense* are vital trees in Ethiopian communities because of their ecological and economic roles, but overharvesting has significantly reduced their populations. Among the 300 documented native tree species in Ethiopia, *C*. *africana* and *S*. *guineense* rank prominently as commercially overexploited taxa to the extent of being endangered [92]. Despite these threats, conservation efforts in the study area remain limited, primarily relying on traditional practices, such as homegarden cultivation, live fencing, and community reforestation. Although their effectiveness is often constrained by the absence of institutionalized governance frameworks, similar practices have been documented in other Ethiopian communities [93]. The integration of WEPs into agroforestry systems (e.g., terracing and fallow land enrichment) demonstrates potential, as evidenced by studies advocating mixed‐crop systems to enhance biodiversity [94].

### 4.9. Novel Ethnobotanical Findings

The Waldeba Monastery’s Arma Dega forest represents a unique ethnobotanical treasure as one of Ethiopia’s last remaining old‐growth woodland ecosystems, supported by continuous monastic stewardship since approximately 490 AD [95]. Yams (*Dioscorea* species) are rich in carbohydrates, dietary fiber, and essential micronutrients, such as potassium, magnesium, and vitamin C, making them valuable food security crops in tropical regions [96]. These species also contain bioactive compounds exhibiting anti‐inflammatory, antioxidant, antimicrobial, antimutagenic, antiobesity, antidiabetic, anticancer (specifically ovarian cancer), menopausal, antiaging, antiasthmatic, diuretic, urinary tract, bladder‐related, rheumatogenic, arthritic, and antispasmodic effects [96, 97]. The findings of this study, along with previous research, underscore the dual role of *Dioscorea* species as nutritional and medicinal resources. However, some species require detoxification because of the presence of toxic compounds, such as dioscorine, highlighting the need for proper processing [67].

## 5. Conclusion and Recommendations

The documented WEPs in the Addi Arkay District play a vital role in food security, nutrition, therapeutic potential, cultural values, and local livelihoods, thereby contributing significantly to income generation and dietary diversification in this drought‐prone region. Indigenous knowledge of WEPs in the study area was strongly influenced by sociodemographic factors, such as nutritional experience, gender, age, and education, as well as socioeconomic factors, such as occupation, which collectively shaped patterns of plant use, access, and knowledge transmission. This interrelationship is crucial for designing context‐specific conservation, education, and food security strategies aligned with local realities. Importantly, the findings revealed that older generations possessed greater WEP knowledge, emphasizing the urgency of preserving and transferring this indigenous ecological knowledge to younger community members to prevent knowledge erosion and sustain cultural continuity. The collection of WEPs from diverse habitats further illustrates the rich biodiversity of the district and reflects the adaptive strategies developed by local communities to cope with food shortages and environmental variability. The varied use of edible plant parts contributes to household food security by providing year‐round dietary options and nutritional diversity, underscoring the crucial role of WEPs in supplementing diets and mitigating seasonal food shortages, particularly in areas where conventional agriculture remains vulnerable to climatic and socioeconomic challenges.

The study found that wide variations in citation frequency and use values reflected strong community preferences, differing levels of species popularity, and varying degrees of cultural importance among WEPs in the local community. These results demonstrate that ethnobotanical knowledge and traditional practices play key roles in shaping species utilization patterns. The preference ranking revealed that *D*. *mespiliformis* was the most favored WEP, reflecting cultural and sensory preferences, whereas DMR highlighted *C. africana* and *D*. *mespiliformis* as multipurpose species used for fuel, construction, food, and fodder. The JSI indicated significant variations in WEP composition across Ethiopian regions, influenced by local ecological and sociocultural factors. Deforestation for agriculture, fuelwood collection, and construction represents the major threats to WEPs in the study area, underscoring the need for sustainable land‐use practices and alternative resource management strategies.

To ensure the sustainable use of threatened WEPs, conservation strategies should integrate both in situ and ex situ approaches that combine traditional ecological knowledge with scientific conservation frameworks. Promoting community awareness and encouraging youth participation in the conservation, documentation, and sustainable utilization of WEPs are also crucial for safeguarding indigenous knowledge, biodiversity, and long‐term food security. Furthermore, incorporating WEP knowledge into community‐based education, school curricula, and local conservation initiatives is essential for strengthening intergenerational knowledge transfer, maintaining cultural continuity, and enhancing food security resilience.

This study was limited to documenting WEPs and associated indigenous knowledge without further phytochemical validation. Future research should therefore focus on proximate composition, antioxidant, and antinutritional factors, as well as pharmacological validation of nutraceutical plants through both *in vitro* and *in vivo* investigations.

## Author Contributions

Worku Misganaw: conceptualization, data collection, formal analysis, writing–original draft, and revision. Getinet Masresha: supervision, botanical identification, critical review, and funding management. Ermias Lulekal: conceptualization, method refinement, and supervision. Asmamaw Alemu: Supervision, critical review, and funding management. Daniel Tadesse: data analysis, supervision, and editing. Worku Misganaw had full access to all of the data sets in this study and takes full responsibility for the integrity of the data and the accuracy of the data analysis.

## Funding

This research was funded by the Research and Technology Transfer Vice President’s Office of the University of Gondar (Postgraduate Strengthening Award; RCSTT 02). Additional support was provided by the IDEA WILD (501c(3)) through the donation of field equipment.

## Disclosure

All the authors have read and approved the final version of the manuscript.

## Ethics Statement

Ethical approval for this study was obtained from the University of Gondar, Department of Biology (Clearance No. 419/2024). 419/2024). Prior to data collection, informed consent was obtained from all participants, and the study was conducted in accordance with the ethical guidelines of the International Society of Ethnobiology (ISE). Due to low literacy levels among participants, verbal informed consent was obtained, and participation was entirely voluntary. The study adhered to internationally recognized ethnobotanical standards, including confidentiality, respect for indigenous knowledge, and compliance with local laws.

## Conflicts of Interest

The authors declare no conflicts of interest.

## Supporting Information

Additional supporting information can be found online in the Supporting Information section.

## Supporting information


**Supporting Information 1** Supporting file 1: Sociodemographic and socioeconomic profile of informants in Addi Arkay District of Ethiopia.

**Supporting Information 2** Supporting file 2: List of wild and semi‐wild edible plants in Addi Arkay District of Ethiopia.

**Supporting Information 3** Supporting file 3: Frequency of citation for WEPs in Addi Arkay District of Ethiopia.

**Supporting Information 4** Supporting file 4: Use value of wild and semi‐wild edible plants in Addi Arkay District of Ethiopia.

## Data Availability

The data supporting the findings of this study are included in the tables, figures, and supporting files.
