# Supplementary material for: Ethnobotanical Study of Wild and Semi‐Wild Edible Plants in Addi Arkay District, Northwestern Ethiopia
Source: ScientificWorldJournal. 2026 Mar 20;2026:6632779. doi: 10.1155/tswj/6632779 (PMC13140352; doi:10.1155/tswj/6632779)
Supplement: Supplementary file 1 — Supporting Information 1 Supporting file 1: Sociodemographic and socioeconomic profile of informants in Addi Arkay District of Ethiopia. [file TSWJ-2026-6632779-s003.docx]

**Supplementary file 1:** Socio-demographic and socio-economic profile of informants in Addi Arkay District of Ethiopia

| **Study Kebeles** |
| --- |

|  | | | **Ali** | **Nebira Fikika** | **Chew Ber** | **Ber Mariyam** | **Arma Dega** | **Agidamiya** | **Teta Ferekwari** | **Semen Teraroch** | **Total** |
| --- | --- | --- | --- | --- | --- | --- | --- | --- | --- | --- | --- |
| GPS coordinates | | | 13°30'30''N  37°44'17''E | 13°22'54''N  37°52'13''E | 13°20'34''N  37°59'02''E | 13°26'53''N  37°59'08''E | 13°30'10''N  37°5016''E | 13°18'52''N  38°07'59''E | 13°19'56''N  38°11'09''E | 13°16'25''N  38°07'42''E | **-** |
| Altitude (m) | | | 1257 m | 1383 m | 1428 m | 1357 m | 1054 m | 2204 m | 2927 m | 3694 m | **-** |
| Agroecology | | | Kolla/low land | Kolla/low land | Kolla/low land | Kolla/low land | Kolla/low land | Woyina Dega/midland | Dega/highland | Wurch/highland  and alpine | 8 |
| NH | | | 1860 | 1451 | 1158 | 1330 | 1540 | 732 | 1007 | 1270 | 10348 |
| NI | |  | 69 | 54 | 43 | 50 | 57 | 27 | 38 | 47 | 385 |
| Informant type | | GI | 64 | 51 | 39 | 46 | 53 | 24 | 34 | 44 | 355 |
|  |  | KI | 5 | 3 | 4 | 4 | 4 | 3 | 4 | 3 | 30 |
| Gender | | M | 42 | 31 | 29 | 32 | 29 | 16 | 22 | 28 | 229 |
|  |  | W | 27 | 23 | 14 | 18 | 28 | 11 | 16 | 19 | 156 |
| Age | | 20-39 | 30 | 26 | 25 | 13 | 21 | 16 | 14 | 21 | 166 |
|  |  | 40-59 | 18 | 14 | 13 | 18 | 26 | 3 | 13 | 15 | 120 |
|  |  | ≥ 60 | 21 | 14 | 5 | 19 | 10 | 8 | 11 | 11 | 99 |
| Education status | | Illiterate | 62 | 50 | 40 | 46 | 38 | 22 | 32 | 36 | 326 |
|  |  | Literate | 7 | 4 | 3 | 4 | 19 | 5 | 6 | 11 | 59 |
| Marital status | | | Ma, Si, Wi | Ma, Si, Wi | Ma, Si, Wi | Ma, Si, Wi, Di | Si, Di | Ma, Si, Wi | Ma, Si, Wi | Ma, Si, Di | - |
| Ethnicity | | | A, T | A, T, Ag | A, T | A, T | A, T, Ag | A, T | A, T, Ag | A, T | - |
| Religion | | | O, Mu | O, Mu | O, Mu | O, Mu | O | O, Mu | O, Mu | O, Mu | - |
| Occupation | | | FA, Me, Em | FA, Me, Em | FA, Em | FA, Me, Em | As | FA, Me, Em, Tw | FA, Me, Em, Tw | FA, Em, Tw | - |
| Income level | Poor < $1000 | | 47 | 32 | 31 | 35 | 57 | 17 | 23 | 28 | 270 |
|  | Medium $1000-$30000 | | 16 | 14 | 10 | 13 | - | 7 | 13 | 16 | 89 |
|  | Rich >$30000 | | 6 | 8 | 2 | 2 | - | 3 | 2 | 3 | 26 |
| Transport access | Accessible | | - | 26 | 18 | 22 | - | - | 7 | 32 | 105 |
|  | Inaccessible | | 69 | 28 | 25 | 28 | 57 | 27 | 31 | 15 | 280 |

**Note: NH =** Number of household, **NI** = Number of interviewees, **Gender** (M = men, F = Women), **Informant type** (GI = General Informant, KI = Key Informant), Marital status (Ma = married, Si = single, Wi = Widowed, D= Divorced), **Ethnicity** (A = Amhara, T = Tigray, Ag = Agew), Religion (O= Orthodox, Mu = Muslim), Occupation (Fa = Farmer, Me = Merchant, Em = Employed (in a formal job), As = Ascetics (Monk/nun), Tw = Two or more occupations, Wealth status (Poor =( < $1000 , Medium =$1000-$10000 , Rich >$10000 ), Transport access (Accessible = road and car, Inaccessible= nor road and car).
