# Supplementary material for: Ethnobotanical Study of Wild and Semi‐Wild Edible Plants in Addi Arkay District, Northwestern Ethiopia
Source: ScientificWorldJournal. 2026 Mar 20;2026:6632779. doi: 10.1155/tswj/6632779 (PMC13140352; doi:10.1155/tswj/6632779)
Supplement: Supplementary file 2 — Supporting Information 2 Supporting file 2: List of wild and semi‐wild edible plants in Addi Arkay District of Ethiopia. [file TSWJ-2026-6632779-s004.docx]

**Supplementary file 2:** List of wild and semi-wild edible plants (WEPs) in Addi Arkay District of Ethiopia

| **Scientific name** | **Family** | **Local Name** | **Habit** | **Part Used** | **Mode of Consumption** | **Harvesting Months** | **Use diversity** | **OE** | **Habitat** | **V. No** |
| --- | --- | --- | --- | --- | --- | --- | --- | --- | --- | --- |
| *Acanthus polystachyus* Delile | Acanthaceae | **Sete kushele** | S | Flower  nectar | Fresh raw, suck nectar in the flower | Sep-Nov | Food, bee forage, firewood, crossfire ceremony animal feed | SF | NF | MW  114/25 |
| *Acanthus sennii* Chiov.*** ^LC^** | Acanthaceae | **Wonde kushele** | S | Flower  nectar | Fresh raw, suck the nectar in the flower | Sep-Nov | Food, bee forage, firewood, crossfire ceremony, animal feed | SF | NF | MW  133/25 |
| *Ampelocissus schimperiana* (Hochst. ex A.Rich.) Planch. | Vitaceae | **Agamshiro** | S | Stem | Peel and eat the fresh raw stem | Mar-May | Food | SF | NF | MW  123/25 |
| *Capparis tomentosa* Lam. | Capparaceae | **Gimero** | S | Fruit | Fresh raw eaten | Sep-Nov | Firewood, bee forage, animal feed, fence, medicine | FF | NF | WM  014/25 |
| *Carissa spinarum* L. | Apocynaceae | **Agam** | S | Fruit | Fresh raw eaten | Mar-May | Food, medicine, fence, fire wood, bee forage | SF | NF | WM  025/25 |
| *Colocasia esculenta* (L.) Schott | Araceae | **Godere** | H | Leaf,  Corm | Fresh cooked as cabbage (leaf), cooked the corm as potatoes | All months | Food, ornamental | SF | Ri | MW  115/25 |
| *Corchorus olitorius* L. | Malvaceae | **Melekuya** | H | Leaf, Stem | Fresh raw eaten (Leaf), cooked (Leaf , young stem) | Jun-Aug | Food, medicine | MD | HG | WM  052/25 |
| *Cordia africana* Lam. | Boraginaceae | **Wanza** | T | Fruit | fresh raw eaten and dry eaten (crush with stone and eat) | Dec-Feb | Food, medicine, fire wood, Furniture, bee forage , farm tools, charcoal, house construction | SF | NFAL  HG | WM  041/25 |
| *Datura stramonium* L. | Solanaceae | **Astenagir** | S | Flower  nectar | Fresh raw, suck nectar in the flower | Jul-Aug | Food, medicine, bee forage | SF | NF  AL HG | WM  015/25 |
| *Dioscorea bulbifera* L. | Dioscoreaceae | **Gimelo** | C | Tuber | Boiled, Peeled, chopped, dried and boiled and eat | Sep-Nov | Food, ration (Quarif)for Waldeba ascetics | MD | NF | MW  116/25 |
| *Dioscorea hispida* Dennst. | Dioscoreaceae | **Tabile** | C | Tuber | Boiled, soaked with water, peeled, chopped, dried, boiled and eat | Sep-Nov | Food, ration (Quarif)for Waldeba ascetics | MD | NF | MW  117/25 |
| *Dioscorea praehensilis* Benth. | Dioscoreaceae | **Sada** | C | Tuber | Boiled, peeled, chopped, dried and boiled and eat | Sep-Nov | Food, ration (Quarif)for Waldeba ascetics | MD | NF | MW  118/25 |
| *Diospyros mespiliformis* Hochst. ex A.DC. | Ebenaceae | **Serkin (aye)** | T | Fruit | Fresh raw eaten | Dec-Feb | Food, tooth brushing, house construction, charcoal, live and dead fence, fire wood | SF | NF  AL HG | WM  053/25 |
| *Dovyalis abyssinica* (A.Rich.) Warb. | Salicaceae | **Koshim** | S | Fruit | Fresh raw eaten | Jun-Aug | Food, medicine, fire wood, bee forage, live and dead fence | SF | NF | WM  100/25 |
| *Ficus sur* Forssk. | Moraceae | **Shola** | T | Fruit | Fresh and dry raw eaten | Dec-Feb | Food, medicine, charcoal, fire wood, furniture | SF | Ri | MW  125/25 |
| *Ficus sycomorus* L. | Moraceae | **Bamba** | T | Fruit | Fresh and dry raw eaten | Dec-Feb | Medicine, food, charcoal, fire wood, furniture | SF | NF  AL | WM  016/25 |
| *Ficus thonningii* Blume | Moraceae | **Chibeha** | T | Fruit | Fresh raw eaten | Dec-Feb | Food, fire wood, charcoal, house construction, animal feed | SF | NF  AL HG | MW  126/25 |
| *Ficus vasta* Forssk. | Moraceae | **Warka** | T | Fruit | Dry raw eaten | Dec-Feb | Food, charcoal, house construction, paste, firewood, animal food | SF | NF  AL | MW  121/25 |
| *Flueggea virosa* (Roxb. ex Willd.) Royle | Phyllanthaceae | **Ayahida** | S | Fruit | Fresh raw eaten | Jul-Aug | Food, firewood, Agricultural tools | FF | NF | MW  119/25 |
| *Gardenia ternifolia* Schumach. & Thonn. | Rubiaceae | **Gambilo** | S | Fruit | Fresh raw eaten | Jun-Aug | Food, medicine, fire wood, charcoal, house construction, animal feed | FF | NF | WM  005/25 |
| *Grewia ferruginea* Hochst. ex A.Rich. | Malvaceae | **Lenkwata** | S | Fruit | Fresh raw eaten | Sep-Nov | Medicine , food, firewood, making basket, washing hair, hive making, stick | SF | NF | WM  028/25 |
| *Mimusops kummel* Bruce ex A.DC. | Sapotaceae | **Kummel (ishe )** | T | Fruit | Fresh raw eaten | Sep-Nov | Food, house construction, fire wood, charcoal | SF | NF  Ri | MW  127/25 |
| *Ocimum grandiflorum* Lam. | Lamiaceae | **Metita** | S | Flower  nectar | Fresh raw, suck the nectar in the flower | Sep-Nov | Food, fire wood, food flavoring | SF | HG | MW  124/25 |
| *Opuntia ficus-indica* (L.) Mill. | Cactaceae | **Beles** | S | Fruit | Fresh raw eaten | Mar-May | Medicine, food | MD | NF | WM  082/25 |
| *Phoenix reclinata* Jacq. | Arecaceae | **Selen** | T | Fruit | Fresh raw eaten | Jun- Aug | Food, ornamental, house construction, teeth toothbrush, rope, mate, basket | SF | NF  Ri | MW  132/25 |
| *Rosa abyssinica* R.Br. ex Lindl. | Rosaceae | **Kega** | S | Fruit | Fresh raw eaten | Mar-May | Food, medicine, live and dead fence | SF | NF | WM  084/25 |
| *Rubus steudneri* Schweinf. | Rosaceae | **Enjori** | S | Fruit | Fresh raw eaten | Mar-May | Food, fire wood | SF | NF | MW  129/25 |
| *Rumex abyssinicus* Jacq. | Polygonaceae | **Mekmoko** | H | Root, Stem | Dry, crush then boil with tea; peel and eat fresh stem | Sep-Nov | Food, medicine, spice for butter | SF | NF | WM  009/25 |
| *Rumex nervosus* Vahl | Polygonaceae | **Embacho** | H | Young  stem | Roasted, piled and then eaten | Sep-Nov | Food, medicine | SF | NF | WM  033/25 |
| *Saba comorensis* (Bojer ex A.DC.) Pichon | Apocynaceae | **Ashama** | C | Fruit | Fresh raw eaten | Sep-Nov | Food, live fence, firewood | SF | NF | MW  120/25 |
| *Searsia glutinosa* (Hochst. ex A.Rich.) Moffett | Anacardiaceae | **Kamuna** | S | Fruit | Fresh raw eaten | Jun-Aug | Food, charcoal, stick, fence, farming material, house construction, fire wood, bee forage | SF | NF | MW  128/25 |
| *Solanum villosum* Mill. | Solanaceae | **Key awute** | H | Fruit | Fresh raw eaten | Sep-Nov | Food | SF | NF  HG | MW  134/25 |
| *Strychnos innocua* Delile | Loganiaceae | **Tinkohaye** | T | Fruit | Fresh raw eaten | Mar-May | Food, firewood | SF | NF  HG | MW  122/25 |
| *Syzygium guineense* (Willd.) DC. | Myrtaceae | **Dokima** | T | Fruit | Fresh raw eaten | Mar-May | Food, house construction, fire wood, charcoal, fence, bee forage | SF | Ri | MW  130/25 |
| *Tamarindus indica* L. | Fabaceae | **Humer** | T | Fruit | Fresh raw, dissolve with water & drink | Dec-Feb | Food, medicine, fodder, timber, firewood, charcoal, ornamental | SF | NF | WM  056/25 |
| *Urtica simensis* Hochst. ex A.Rich. ***^LC^** | Urticaceae | **Sama** | H | Leaf | Fresh cooked as stew | Jun-Aug | Food, medicine | SF | NF | WM  110/25 |
| *Vachellia abyssinica* (Hochst. ex Benth.) Kyal. & Boatwr | Fabaceae | **Bazira girar** | T | Gum | Fresh raw, chewing | All months | Medicine, food, dead fence, charcoal, fire wood, farm tools, bee forage, animal feed | SF | NF | WM  097/25 |
| *Vachellia seyal* var. *fistula* (Schweinf.) Kyal. & Boatwr. | Fabaceae | **Qontire girar** | T | Gum | Fresh raw, chewing | All months | Food, dead fence, charcoal, fire wood, farm tools, bee forage, animal feed | SF | NF | MW  113/25 |
| *Vangueria madagascariensis* J.F.Gmel. | Rubiaceae | **Embua baria/Guramile** | S | Fruit | Fresh raw eaten | Dec-Feb | Food, firewood | SF | Ri | MW  131/25 |
| *Ximenia americana* L. | Olacaceae | **Enkoy** | S | Fruit | Fresh raw eaten | Jun-Aug | Food, medicine, firewood, charcoal | SF | NF | WM  058/25 |
| *Ziziphus mucronata* Willd. | Rhamnaceae | **Abetere** | T | Fruit | Fresh raw eaten | Dec-Feb | Food, fire wood, live and dead fence | SF | NF | WM  059/25 |
| *Ziziphus spina-christi* (L.) Willd. | Rhamnaceae | **Gaba** | S | Fruit | Fresh raw eaten | Dec-Feb | Food, medicine, live and dead fence ,fire wood, charcoal, farm material | SF | NF | WM  061/25 |

**Note:** (***** = endemic plants, LC= Least Concern, m.a.s.l = meter above sea level, OE = Occasions of eating, SF = Supplementary food, MD = main dish, FF= famine food, HG = Home Garden, Ri = Riverine, AL = Arable Land, NF = Natural Forest, V. No = Voucher number)
