# Supplementary material for: Ethnobotanical Study of Wild and Semi‐Wild Edible Plants in Addi Arkay District, Northwestern Ethiopia
Source: ScientificWorldJournal. 2026 Mar 20;2026:6632779. doi: 10.1155/tswj/6632779 (PMC13140352; doi:10.1155/tswj/6632779)
Supplement: Supplementary file 3 — Supporting Information 3 Supporting file 3: Frequency of citation for WEPs in Addi Arkay District of Ethiopia. [file TSWJ-2026-6632779-s002.docx]

**Supplementary file 3**: Frequency of citation for WEPs in Addi Arkay District of Ethiopia

| **No** | **Scientific name** | **No. of informant citations** | **Percentage (%)** |
| --- | --- | --- | --- |
| 1 | *Diospyros mespiliformis* Hochst. ex A.DC. | 321 | 83.38 |
| 2 | *Ziziphus spina-christi* (L.) Willd. | 308 | 80.00 |
| 3 | *Cordia africana* Lam. | 297 | 77.14 |
| 4 | *Carissa spinarum* L. | 284 | 73.77 |
| 5 | *Ximenia americana* L. | 267 | 69.35 |
| 6 | *Syzygium guineense* (Willd.) DC. | 249 | 64.68 |
| 7 | *Ficus vasta* Forssk. | 235 | 61.04 |
| 8 | *Ficus sycomorus* L. | 234 | 60.78 |
| 9 | *Mimusops kummel* Bruce ex A.DC. | 221 | 57.40 |
| 10 | *Grewia ferruginea* Hochst. ex A.Rich. | 204 | 52.99 |
| 11 | *Ficus sur* Forssk. | 192 | 49.87 |
| 12 | *Searsia glutinosa* (Hochst. ex A.Rich.) Moffett | 160 | 41.56 |
| 13 | *Opuntia ficus-indica* (L.) Mill. | 156 | 40.52 |
| 14 | *Ficus thonningii* Blume | 131 | 34.03 |
| 15 | *Tamarindus indica* L. | 130 | 33.77 |
| 16 | *Rosa abyssinica* R.Br. ex Lindl. | 125 | 32.47 |
| 17 | *Ziziphus mucronata* Willd. | 115 | 29.87 |
| 18 | *Dovyalis abyssinica* (A.Rich.) Warb. | 102 | 26.49 |
| 19 | *Vangueria madagascariensis* J.F.Gmel. | 100 | 25.97 |
| 20 | *Dioscorea praehensilis* Benth. | 86 | 22.34 |
| 21 | *Phoenix reclinata* Jacq. | 83 | 21.56 |
| 22 | *Strychnos innocua* Delile | 79 | 20.52 |
| 23 | *Rumex abyssinicus* Jacq. | 75 | 19.48 |
| 24 | *Rumex nervosus* Vahl | 70 | 18.18 |
| 25 | *Flueggea virosa* (Roxb. ex Willd.) Royle | 69 | 17.92 |
| 26 | *Dioscorea hispida* Dennst. | 65 | 16.88 |
| 27 | *Rubus steudneri* Schweinf. | 64 | 16.62 |
| 28 | *Saba comorensis* (Bojer ex A.DC.) Pichon | 62 | 16.10 |
| 29 | *Dioscorea bulbifera* L. | 59 | 15.32 |
| 30 | *Corchorus olitorius* L. | 54 | 14.03 |
| 31 | *Gardenia ternifolia* Schumach. & Thonn. | 52 | 13.51 |
| 32 | *Ocimum grandiflorum* Lam. | 49 | 12.73 |
| 33 | *Solanum villosum* Mill. | 48 | 12.47 |
| 34 | *Vachellia abyssinica* (Hochst. ex Benth.) Kyal. & Boatwr | 46 | 11.95 |
| 35 | *Acanthus polystachyus* Delile | 43 | 11.17 |
| 36 | *Vachellia seyal* var. fistula (Schweinf.) Kyal. & Boatwr. | 42 | 10.91 |
| 37 | *Acanthus sennii* Chiov. | 40 | 10.39 |
| 38 | *Urtica simensis* Hochst. ex A.Rich. | 34 | 8.83 |
| 39 | *Capparis tomentosa* Lam. | 31 | 8.05 |
| 40 | *Ampelocissus schimperiana* (Hochst. ex A.Rich.) Planch. | 30 | 7.79 |
| 41 | *Colocasia esculenta* (L.) Schott | 19 | 4.94 |
| 42 | *Datura stramonium* L. | 16 | 4.16 |
