# Supplementary material for: Ethnobotanical Study of Wild and Semi‐Wild Edible Plants in Addi Arkay District, Northwestern Ethiopia
Source: ScientificWorldJournal. 2026 Mar 20;2026:6632779. doi: 10.1155/tswj/6632779 (PMC13140352; doi:10.1155/tswj/6632779)
Supplement: Supplementary file 4 — Supporting Information 4 Supporting file 4: Use value of wild and semi‐wild edible plants in Addi Arkay District of Ethiopia. [file TSWJ-2026-6632779-s001.docx]

**Supplementary file 4:** Use vale of wild and WEPs in Addi Arkay District of Ethiopia

| **No** | **Scientific name** | **Uis** | **ns** | **Uvs** |
| --- | --- | --- | --- | --- |
| 1 | *Diospyros mespiliformis* Hochst. ex A.DC. | 29 | 30 | 0.97 |
| 2 | *Cordia africana* Lam. | 28 | 30 | 0.93 |
| 3 | *Syzygium guineense* (Willd.) DC. | 25 | 30 | 0.83 |
| 4 | *Ficus vasta* Forssk. | 24 | 30 | 0.80 |
| 5 | *Ziziphus spina-christi* (L.) Willd. | 23 | 30 | 0.77 |
| 6 | *Vachellia abyssinica* (Hochst. ex Benth.) Kyal. & Boatwr | 22 | 30 | 0.73 |
| 7 | *Carissa spinarum* L. | 21 | 30 | 0.70 |
| 8 | *Tamarindus indica* L. | 19 | 30 | 0.63 |
| 9 | *Ficus sycomorus* L. | 18 | 30 | 0.60 |
| 10 | *Grewia ferruginea* Hochst. ex A.Rich. | 15 | 30 | 0.50 |
| 11 | *Ficus sur* Forssk. | 14 | 30 | 0.47 |
| 12 | *Ficus thonningii* Blume | 14 | 30 | 0.47 |
| 13 | *Mimusops kummel* Bruce ex A.DC. | 14 | 30 | 0.47 |
| 14 | *Dioscorea hispida* Dennst. | 13 | 30 | 0.43 |
| 15 | *Searsia glutinosa* (Hochst. ex A.Rich.) Moffett | 13 | 30 | 0.43 |
| 16 | *Dioscorea bulbifera* L. | 12 | 30 | 0.40 |
| 17 | *Opuntia ficus-indica* (L.) Mill. | 12 | 30 | 0.40 |
| 18 | *Dovyalis abyssinica* (A.Rich.) Warb. | 11 | 30 | 0.37 |
| 19 | *Dioscorea praehensilis* Benth. | 10 | 30 | 0.33 |
| 20 | *Ziziphus mucronata* Willd. | 10 | 30 | 0.33 |
| 21 | *Phoenix reclinata* Jacq. | 9 | 30 | 0.30 |
| 22 | *Vangueria madagascariensis* J.F.Gmel. | 8 | 30 | 0.27 |
| 23 | *Ximenia americana* L. | 7 | 30 | 0.23 |
| 24 | *Flueggea virosa* (Roxb. ex Willd.) Royle | 6 | 30 | 0.20 |
| 25 | *Gardenia ternifolia* Schumach. & Thonn. | 6 | 30 | 0.20 |
| 26 | *Strychnos innocua* Delile | 5 | 30 | 0.17 |
| 27 | *Vachellia seyal* var. fistula (Schweinf.) Kyal. & Boatwr. | 5 | 30 | 0.17 |
| 28 | *Rosa abyssinica* R.Br. ex Lindl. | 4 | 30 | 0.13 |
| 29 | *Rubus steudneri* Schweinf. | 3 | 30 | 0.10 |
| 30 | *Rumex abyssinicus* Jacq. | 3 | 30 | 0.10 |
| 31 | *Rumex nervosus* Vahl | 3 | 30 | 0.10 |
| 32 | *Urtica simensis* Hochst. ex A.Rich. | 3 | 30 | 0.10 |
| 33 | *Acanthus polystachyus* Delile | 2 | 30 | 0.07 |
| 34 | *Acanthus sennii* Chiov. | 2 | 30 | 0.07 |
| 35 | *Corchorus olitorius* L. | 2 | 30 | 0.07 |
| 36 | *Datura stramonium* L. | 2 | 30 | 0.07 |
| 37 | *Ocimum grandiflorum* Lam. | 2 | 30 | 0.07 |
| 38 | *Ampelocissus schimperiana* (Hochst. ex A.Rich.) Planch. | 1 | 30 | 0.03 |
| 39 | *Capparis tomentosa* Lam. | 1 | 30 | 0.03 |
| 40 | *Colocasia esculenta* (L.) Schott | 1 | 30 | 0.03 |
| 41 | *Saba comorensis* (Bojer ex A.DC.) Pichon | 1 | 30 | 0.03 |
| 42 | *Solanum villosum* Mill. | 1 | 30 | 0.03 |

(Uvs= use value of species S, Uis = the number of uses of species S according to key informant i, and ns = the total number key informants interviewed)
